# Interpretable photoplethysmography-based machine-learning model for noninvasive assessment of systolic blood pressure

**DOI:** 10.3389/fphys.2026.1779262

**Published:** 2026-06-01

**Authors:** Chin-Nan Lin, Chun-Cheng Wang, Pei-Chun Chao, Jen-Jyh Lin, Chih-Ping Chang, Hsin-Min Lu, Po-Wei Lin, Po-Yen Ko

**Affiliations:** 1 China Medical University, Taichung, Taiwan; 2 Department of Chinese Medicine, Dalin Tzu Chi Hospital, The Buddhist Tzu Chi Medical Foundation, Chiayi, Taiwan; 3 Division of Cardiology, Department of Medicine, China Medical University Hospital, Taichung, Taiwan

**Keywords:** hypertension, logistic models, machine-learning, photoplethysmography, remote sensing technology, wearable electronic devices

## Abstract

**Background:**

Hypertension significantly contributes to cardiovascular morbidity and mortality but is often underdiagnosed and poorly controlled, as intermittent cuff-based measurements provide only discrete readings and miss short-term vascular dynamics. Photoplethysmography (PPG), a noninvasive optical technique tracking pulsatile blood-volume changes, offers a practical, low-cost, wearable-compatible alternative capable of capturing temporal and morphological waveform patterns. Unlike dual-sensor or calibration-dependent systems, single-site PPG can extract indices linked to arterial stiffness, compliance, and wave reflection, providing physiologically interpretable vascular information. Advances in signal processing and interpretable machine learning have shifted PPG analysis from descriptive morphology to feature-driven clinical prediction. Integrating waveform metrics—such as systolic amplitude, contour sharpness, and harmonic energy distribution—with demographic variables enables transparent modeling of blood-pressure status.

**Objective:**

To develop an interpretable machine-learning model for classifying systolic blood pressure (SBP) status using physiological and morphological features derived from a single PPG signal.

**Methods:**

We analyzed 860 participants and extracted waveform-derived and clinical features from 90-second fingertip PPG recordings. A LASSO-regularized logistic regression model with fivefold cross-validation was used for feature selection and classification, and model interpretability was assessed using SHAP values.

**Results:**

The final model achieved an AUC of 0.83 (95% CI 0.79–0.87), an F1-score of 0.83, and a Cohen’s d of 1.39. Five predictors were retained: age, BMI, P1 amplitude, waveform sharpness (1_10), and harmonic ratio (H2/H1). Higher P1, greater waveform sharpness, and higher BMI were positively associated with elevated SBP, whereas H2/H1 was negatively associated.

**Conclusion:**

An interpretable PPG-based model using physiologically meaningful features can distinguish hypertensive from normotensive individuals and may serve as a practical digital screening tool for elevated systolic blood pressure.

## 1 Introduction

Hypertension remains one of the most pressing global health challenges, affecting an estimated 1.4 billion individuals, or over 31% of the world’s population as assessed in 2010 (Mills et al., 2016). Despite its high prevalence, fewer than half of affected patients receive appropriate diagnosis or treatment, and nearly two-thirds remain unaware of their condition (Egan et al., 2019). This silent epidemic produces substantial clinical consequences, contributing to cardiovascular disease, stroke, chronic kidney disease, and other life-threatening complications (Lawes et al., 2008). Contemporary clinical guidelines specify that diagnostic and management thresholds for blood pressure (BP) are more stringent than in the past. Hypertension is currently defined as an office systolic blood pressure (SBP) ≥140 mmHg or diastolic blood pressure (DBP) ≥90 mmHg, while values between SBP 120–139 mmHg or DBP 70–89 mmHg are categorized as elevated BP (Badila, 2024). Recent outcome trials have shown that achieving stricter control, with SBP reduced to approximately 120 mmHg, can further decrease cardiovascular risk (Liu et al., 2024). These findings illustrate a global move toward lower treatment targets, emphasizing the importance of optimal blood-pressure control for cardiovascular protection. Nevertheless, many individuals remain unaware of their hypertensive status and therefore receive no treatment (Blood Pressure Lowering Treatment Trialists, C, 2021), leaving them exposed to avoidable long-term risk. The development of accessible detection technology capable of identifying unrecognized hypertension early could substantially improve public health outcomes. By enabling timely diagnosis and treatment initiation, such tools could help reduce chronic cardiovascular complications worldwide.

Photoplethysmography (PPG), a noninvasive optical method that measures pulsatile blood-volume changes in the microvascular bed, has emerged as a promising modality for early hypertension detection (Pankaj et al., 2023). Widely used PPG-based indices, including pulse transit time (PTT) and pulse-wave velocity (PWV), have been extensively studied for BP estimation. However, the accuracy of PWV as measured via PPG can be influenced by physiological factors such as local changes in the vascular tone of muscular arteries. Furthermore, these indices usually require dual-sensor configurations, such as simultaneous ECG and PPG acquisition. This fact, together with repeated calibration, limits their scalability for use in population screening (Esmaelpoor et al., 2020). In contrast, single-site pulse-wave analysis allows extraction of timing and morphological features directly from one PPG waveform, providing a practical and low-cost solution for blood-pressure assessment in clinical and wearable settings. Importantly, the strength of this approach lies not only in single-sensor acquisition but also in the integration of interpretable feature extraction with machine-learning classification.

Recent advances in artificial intelligence (AI) and machine learning (ML) have led to increasing interest in their application to hypertension, particularly for risk prediction, continuous monitoring, and wearable-based blood-pressure assessment (Khamissi et al., 2025). These approaches are especially relevant in hypertension, where blood-pressure regulation is influenced by multiple interacting factors, including physiological, behavioral, and environmental components, making conventional models difficult to fully capture (Varzideh et al., 2026).

At the same time, cuffless blood-pressure measurement based on pulse-wave analysis has rapidly developed in recent years. However, despite promising results, important limitations remain, including reliance on calibration, concerns regarding validation methodology, and uncertainty in real-world performance (Mukkamala et al., 2025). These issues highlight that, in addition to predictive accuracy, methodological rigor and clinical interpretability are essential for meaningful translation into practice (Varzideh et al., 2026).

From a broader perspective, AI-driven precision medicine has emphasized the use of physiological and wearable-derived data to identify clinically relevant digital biomarkers and support individualized assessment (Ahmed and Alam, 2026). However, many high-performing ML models remain difficult to interpret from a physiological standpoint, which may limit their clinical adoption. Therefore, there is a need for approaches that retain the advantages of data-driven modeling while preserving clear physiological meaning and interpretability, particularly in physiologically grounded signal analysis.

Recent work, including studies from our group and others (Gonzalez et al., 2023; Lin et al., 2025; Mohammadi et al., 2025), has demonstrated the physiological interpretability and diagnostic potential of PPG-derived time- and frequency-domain features in real-world datasets. These findings support the potential of PPG-based approaches for noninvasive cardiovascular assessment and BP classification. Building on this foundation, the present study aims to develop and validate a rapid, interpretable, single-sensor machine-learning framework to distinguish hypertensive (SBP ≥140 mmHg) from normotensive (SBP ≤120 mmHg) individuals. Using the least absolute shrinkage and selection operator (LASSO) regression, we identified five key predictors, age, body-mass index (BMI), P1, 1_10, and H2/H1, from an initial pool of thirty waveform and clinical variables. These predictors were subsequently evaluated across multiple supervised classifiers, including logistic regression, Random Forest, XGBoost, support-vector machine, and k-nearest neighbors, to benchmark predictive performance and generalizability.

Overall, this study aims to develop a streamlined, interpretable, and computationally efficient machine-learning framework for population-level hypertension screening using physiologically meaningful features derived from PPG waveforms. Furthermore, the proposed framework is designed to link physiological signal analytics with practical digital health deployment.

## 2 Methods

### 2.1 Study design and recruitment

This cross-sectional study was conducted at China Medical University Hospital (Taichung, Taiwan). We enrolled participants consecutively from hospital cardiology outpatient clinics. Eligible participants included adults aged 20 years or older who provided written informed consent and had remained seated at rest for at least 10 minutes prior to measurement. Exclusion criteria included: (1) documented arrhythmias (e.g., atrial fibrillation); (2) presence of a permanent pacemaker; (3) inter-measurement interval < 15 seconds; (4) ongoing maintenance dialysis; (5) severe peripheral arterial disease; (6) structural deformities of the fingers or wrists that may impair sensor placement; (7) incomplete or excessively noisy PPG recordings after preprocessing; and (8) PPG segments with a signal-quality index (SQI) below 0.2%, since this indicates an inadequate pulsatile-to-static component ratio.

The study protocol was reviewed and approved by the Research Ethics Committee III of China Medical University Hospital (Approval No. CMUH114-REC3-111). All participants provided written informed consent in accordance with the Declaration of Helsinki.

**Figure 1:**
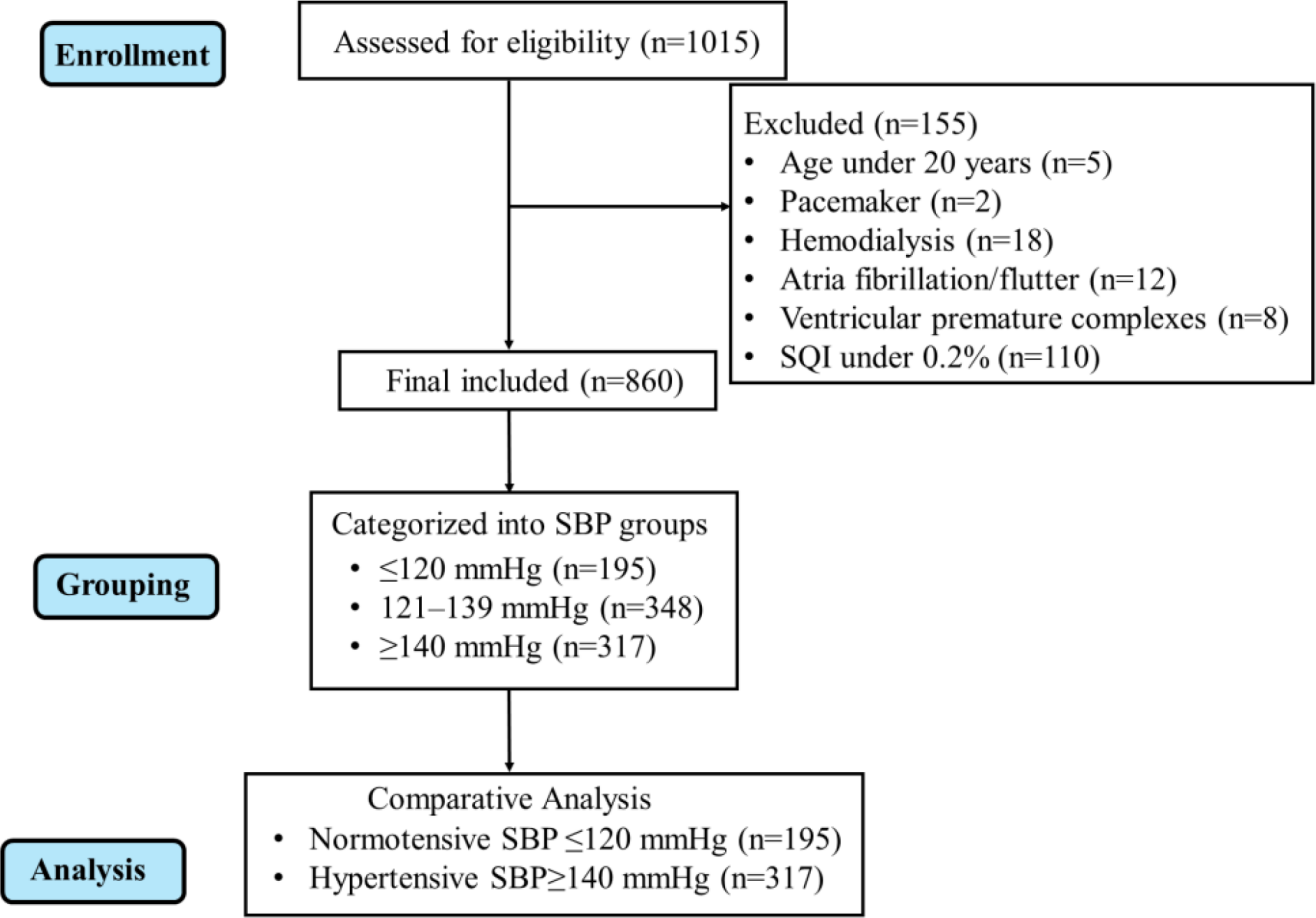
CONSORT-style flow diagram illustrating the participant pathway from enrollment through analysis. The figure details the number of individuals screened, excluded based on clinical criteria or insufficient PPG quality (SQI< 0.2%), and the final cohort stratification into SBP categories (≤120 mmHg and ≥140 mmHg) used for machine-learning model training and evaluation.

The overall participant flow, after enrollment and quality control, is illustrated in **Figure 1**. This diagram summarizes subject recruitment, exclusion criteria, signal-quality filtering, and final stratification into systolic blood-pressure groups (≤120 mmHg and ≥140 mmHg). Participants with intermediate SBP values (121–139 mmHg) were excluded from the classification analysis to ensure clear separation between normotensive and hypertensive groups. This workflow was designed to ensure that only physiologically valid and noise-free PPG segments (SQI ≥0.2%) were included in the final machine-learning analysis.

### 2.2 BP measurement and grouping

BP was measured using a validated automated oscillometric sphygmomanometer (HBP-9030, Omron Healthcare Co., Ltd., Kyoto, Japan) during routine outpatient assessment. Participants were seated comfortably with the right arm supported at heart level and rested for at least 10 minutes prior to measurement. Two BP readings were obtained at one-minute intervals, and the mean values of SBP and DBP were used for analysis, in accordance with current recommendations to base office BP on the average of multiple measurements to reduce variability and improve accuracy (Writing Committee, M, 2025). This measurement protocol is consistent with that used in our previous study (Lin et al., 2025), in which the same device and procedure were applied under comparable clinical conditions. These cuff-based BP measurements served as the clinical reference standard for all subsequent analyses, including feature selection and machine-learning model development.

Participants were stratified into systolic blood pressure categories according to established clinical thresholds. For the primary classification task, individuals with SBP ≤120 mmHg were defined as the normotensive group, whereas those with SBP ≥140 mmHg were defined as the hypertensive group. Participants with intermediate SBP values (121–139 mmHg) were excluded to ensure clear separation between groups and reduce classification ambiguity.

This categorization is consistent with widely accepted clinical guidelines for BP classification, including the Seventh Report of the Joint National Committee (JNC 7). Given that arterial pulse waveforms primarily reflect hemodynamic changes during systole, SBP was used as the primary variable for group definition in this study.

### 2.3 Signal processing and quality control

Overall, the PPG acquisition and preprocessing pipeline used in this study followed a framework described in a previous study from our lab (Lin et al., 2025). However, here we added new methodological refinements to improve signal-quality control.

Next, PPG signals were recorded using a MAX30102 optical sensor (Maxim Integrated, USA) connected to a Raspberry Pi 5 (Raspberry Pi Foundation, UK) through the I²C communication interface. The sampling rate was set at 500Hz, and continuous 90-second recordings were obtained from the left index fingertip under conditions where ambient light was minimized to reduce optical interference.

Raw signals were processed with a band-pass FIR filter (0.5–8 Hz) using a Kaiser window. For baseline correction, a 1.5-second moving-average trend was computed and subtracted from the filtered signal to remove low-frequency baseline drift while preserving beat-to-beat pulsatile components. The window length was chosen to exceed the duration of a single cardiac cycle under resting conditions, thereby capturing slow baseline variations rather than waveform morphology.

The PPG signals were obtained using the MAX30102 optical sensor, which integrates dual-wavelength LEDs consisting of a red LED (approximately 660 nm) and a near-infrared LED (approximately 880 nm) for reflective PPG measurement. In the present study, the infrared channel was primarily used for waveform analysis due to its superior signal stability and deeper tissue penetration. To ensure signal fidelity, we calculated a signal quality index (SQI) as the ratio of the alternating-current (AC) to the direct-current (DC) component of the infrared (IR) waveform; this relationship is shown in the following equation:


$$SQI\;=\;\frac{AC}{DC}\;\times \;100\%$$

Here, AC is defined as the amplitude of the pulsatile component of the IR signal (i.e., max(filtered_IR) − min(filtered_IR)), and DC represents the baseline component, calculated as the mean value of the raw IR signal (mean(raw_IR)).

This ratio is conceptually equivalent to the perfusion index (PI) used in the PPG literature, which quantifies the strength of the pulsatile (AC) component relative to the baseline (DC) and is widely applied to assess signal reliability and tissue perfusion (Park et al., 2021). Recent studies have shown that PI, defined as the AC/DC ratio, can serve effectively as a proxy for signal quality in wearable PPG systems, particularly in low-perfusion or motion-prone settings where it has been included among validated wrist-based signal-quality metrics (Charlton et al., 2025).

Notably, the physiological range of PI has been reported to span widely (approximately 0.2%–20%), with values known to be highly skewed and dependent on measurement conditions, sensor configuration, and individual vascular characteristics (Elshal et al., 2021; Park et al., 2021). This variability suggests that no universal cutoff exists and supports the use of study-specific thresholds.

Segments with SQI<0.2% were excluded because the low pulsatile-to-static ratio often reflects insufficient peripheral perfusion or substantial motion artifacts (Boonya-Ananta et al., 2021). Signals with SQI greater than 0.2% were retained as high-quality waveforms for feature extraction, in agreement with prior work that optimized sensor performance by maximizing the AC/DC ratio (Scardulla et al., 2023). In addition, preliminary analysis of our dataset indicated that segments below this threshold were frequently associated with unstable waveform morphology and reduced feature reproducibility. Therefore, 0.2% was selected as a conservative lower bound to ensure reliable physiological interpretation.

Systolic peaks were identified as local maxima of the filtered waveform after derivative-based zero-crossing verification, following fiducial definitions (Elgendi et al., 2013). Beat-to-beat intervals were computed from consecutive systolic peaks and then used for pulse-rate-variability (PRV) analysis. All measurements were collected under resting outpatient conditions with participants seated and motionless to minimize motion artifacts. The PPG acquisition device incorporated a patented (Taiwan Utility Model No. 114206342) noise-suppression and signal-stabilization design that supported stable waveform acquisition in routine clinical environments.

### 2.4 Feature extraction

We then extracted a comprehensive set of 19 PPG-derived features from each 90-second recording to characterize waveform morphology, timing, and autonomic modulation; this protocol followed previously validated feature definitions (Lin et al., 2025). In brief, these features comprised 13 time-domain features and 6 frequency-domain parameters. Feature extraction was performed on a beat-by-beat basis. Individual pulse cycles were first identified from the filtered infrared PPG waveform, and morphological landmarks were subsequently detected within each pulse. These landmarks included the systolic peak (P1), diastolic peak (P2), valley point (V), and the dicrotic notch, which were automatically identified from the filtered signal.

Time-domain features captured both morphological and rhythmic characteristics of each pulse cycle. The derived indices included systolic and diastolic times (Ts, Td), the peak-time difference (ΔT), systolic and diastolic slopes (Ss, Ds), pulse area, and the systole-to-diastole ratio (Ts/Td). In addition, beat-to-beat variability metrics were calculated to characterize temporal dynamics, including the standard deviation of normal-to-normal intervals (SDNN), root mean square of successive differences (RMSSD), and the percentage of successive intervals differing by more than 50 ms (pNN50). Pulse rate was calculated from the mean pulse interval.

To further quantify waveform morphology, six waveform-sharpness parameters (1_10, 1_8, 1_6, 1_5, 1_3, and 1_2) were computed to describe the contour and sharpness of the PPG waveform. Each parameter reflects the normalized horizontal width of the waveform at fractional height levels of the systolic peak, thereby characterizing the geometric sharpness and steepness of the systolic upstroke. These parameters were defined and validated in our previous work (Khamissi et al., 2025) and were retained for feature selection and subsequent machine-learning analysis.

Frequency-domain features were extracted using Fast Fourier Transform (FFT) in combination with Welch’s method for power spectral density estimation. Harmonic power ratios (H1–H4) were calculated to represent the fundamental and higher-order harmonic energy components of the PPG waveform. In addition, autonomic-related variability indices were derived from the pulse rate variability (PRV) spectrum, including low-frequency power (LF: 0.04–0.15 Hz), high-frequency power (HF: 0.15–0.40 Hz), and the LF/HF ratio, which are commonly used indicators of sympathovagal balance.

Finally, feature values obtained from individual pulse cycles were averaged across stable signal segments that were prescreened using a signal quality index (SQI ≥ 0.2%). Segments with irregular pulse intervals, distorted waveform morphology, or low signal quality were excluded to minimize the influence of ectopic beats or rhythm disturbances. In addition, subjects with documented arrhythmias (e.g., atrial fibrillation or ventricular premature complexes) were excluded based on clinical records. This procedure ensured that only physiologically consistent and stable waveforms contributed to the final feature set used for statistical analysis and model development.

### 2.5 Model development

To identify the most informative predictors of SBP, we applied a LASSO-logistic regression with fivefold cross-validation to all 30 candidate variables (i.e., the 19 PPG-derived and 11 auxiliary clinical or derived metrics). Machine learning was used not only to confirm univariable statistical differences, but to evaluate the joint classification performance of multiple correlated predictors. This approach enables the identification of complementary contributions among demographic and waveform-derived features within a multivariable framework. The LASSO framework applies an L1-regularization penalty that shrinks redundant or low-impact variables toward zero, thereby reducing overfitting and producing a concise, parsimonious, and interpretable subset of features. This also improves model parsimony and reduces the risk of overfitting in the presence of correlated predictors.

In contrast to conventional regression models that retain all predictors, LASSO performs embedded feature selection by shrinking less informative coefficients to zero, thereby improving interpretability and model robustness.

Five key predictors were retained after feature selection: age, BMI, waveform-sharpness index (1_10), systolic amplitude (P1), and harmonic ratio (H2/H1). Together, these features provided the strongest discrimination between normotensive (SBP ≤120 mmHg) and hypertensive (SBP ≥140 mmHg) participants.

The final model used a LASSO-regularized logistic regression trained under fivefold stratified cross-validation. Comparative classifiers, including Random Forest, XGBoost, support-vector machine (SVM-RBF), and k-nearest neighbors (KNN), were evaluated to benchmark performance. All comparative models were implemented using the default hyperparameter settings provided by the scikit-learn library to ensure consistent baseline benchmarking.

Model discrimination was quantified using the area under the receiver-operating-characteristic curve (AUC), average precision (AP), accuracy, F1-score, and Cohen’s d effect size, with 95% confidence intervals obtained by 2,000 bootstrap resamples.

To enhance interpretability, Shapley Additive exPlanations (SHAP) were applied to the LASSO model to examine each feature’s marginal contribution to hypertension prediction. Positive SHAP values indicated associations with elevated SBP, whereas negative SHAP values reflected characteristics more consistent with normotensive physiology.

### 2.6 Statistical analyses

All analyses were performed in Python version 3.10. We used *scikit-learn* for model development and SHAP for interpretability assessment. Model performance metrics included AUC, AP, accuracy, F1-score, and Cohen’s d as a measure of effect size. All metrics were reported with 95% confidence intervals based on 2,000 bootstrap resamples. All two-tailed p-values< 0.05 were considered statistically significant.

## 3 Results

### 3.1 Baseline characteristics stratified by SBP

The baseline characteristics of participants stratified by SBP are summarized in **Table 1**. Participants in the SBP ≥140 mmHg group were significantly older than those in the SBP ≤120 mmHg group, with a median age of 70 years (IQR 62–76) compared with 62 years (IQR 55–72) (p< 0.001). The proportion of males was similar between groups (54.6% vs. 53.8%, p = 0.945).

**Table 1:** Baseline characteristics of participants stratified by SBP level.

Variable	SBP ≤120	SBP ≥140	*p-value*
Basic information			
Age (years)	62.00 (55.00–72.00)	70.00 (62.00–76.00)	<0.001
Male sex	105 (53.8%)	173 (54.6%)	0.945
BMI	24.56 (21.72–27.16)	25.25 (23.31–28.93)	<0.001
Comorbidities			
CKD	40 (20.5%)	69 (21.8%)	0.822
DM	61 (31.3%)	91 (28.7%)	0.603
CAD	59 (30.3%)	109 (34.4%)	0.385
COPD/asthma	6 (3.1%)	5 (1.6%)	0.411
Medication			
Statin	110 (56.7%)	189 (60.0%)	0.753
CCB	96 (49.5%)	141 (44.8%)	0.575
ACEI/ARB	98 (50.5%)	171 (54.3%)	0.700
α-blocker	14 (7.2%)	19 (6.0%)	0.858
β-blocker	96 (49.5%)	126 (40.0%)	0.110
Thiazide	20 (10.3%)	26 (8.3%)	0.724

Body mass index (BMI) was also higher in the elevated SBP group (25.25 kg/m² [IQR 23.31–28.93]) than in the lower SBP group (24.56 kg/m² [IQR 21.72–27.16], p< 0.001). The prevalence of major cardiovascular comorbidities, including chronic kidney disease, diabetes mellitus, coronary artery disease, and COPD/asthma, did not differ significantly between the two groups (all p > 0.05).

Similarly, the use of cardiovascular medications, including statins, ACEI/ARB, calcium channel blockers, α-blockers, β-blockers, and thiazide diuretics, was comparable between SBP strata (all p > 0.05), although β-blocker use appeared numerically lower in the SBP ≥140 mmHg group (40.0% vs. 49.5%). Overall, the two SBP groups exhibited broadly similar clinical profiles, with age and BMI being the only variables showing statistically significant differences.

### 3.2 Model performance

Among the five classifiers evaluated using fivefold cross-validation, the LASSO-regularized logistic regression model achieved the strongest discrimination for distinguishing participants with SBP ≥140 mmHg from those ≤120 mmHg. As summarized in **Table 2**, the LASSO model produced an AUC of 0.831 (95% CI 0.794–0.868) and an AP of 0.865, with an overall accuracy of 0.79 and an F1 score of 0.83. The corresponding Cohen’s d effect size was 1.39 (95% CI 1.16–1.64), thereby reflecting strong separation between the predicted probabilities of the two SBP groups.

**Table 2:** Class-wise classification performance of the LASSO-regularized logistic regression model.

SBP group	Precision	Recall	F1-score	Support
≤120 mmHg	0.72	0.74	0.73	195
≥140 mmHg	0.84	0.82	0.83	317
Macro average	0.78	0.78	0.78	–
Weighted average	0.79	0.79	0.79	512

Metrics were obtained from fivefold cross-validation to identify individuals with SBP ≥140 mmHg versus ≤120 mmHg. Overall, the model showed strong discrimination and calibration accuracy, yielding an AUC of 0.831 (95% CI 0.794–0.868), an AP of 0.865, and a Cohen’s d value of 1.39, indicating a large separation between the two SBP groups.

**Figure 2:**
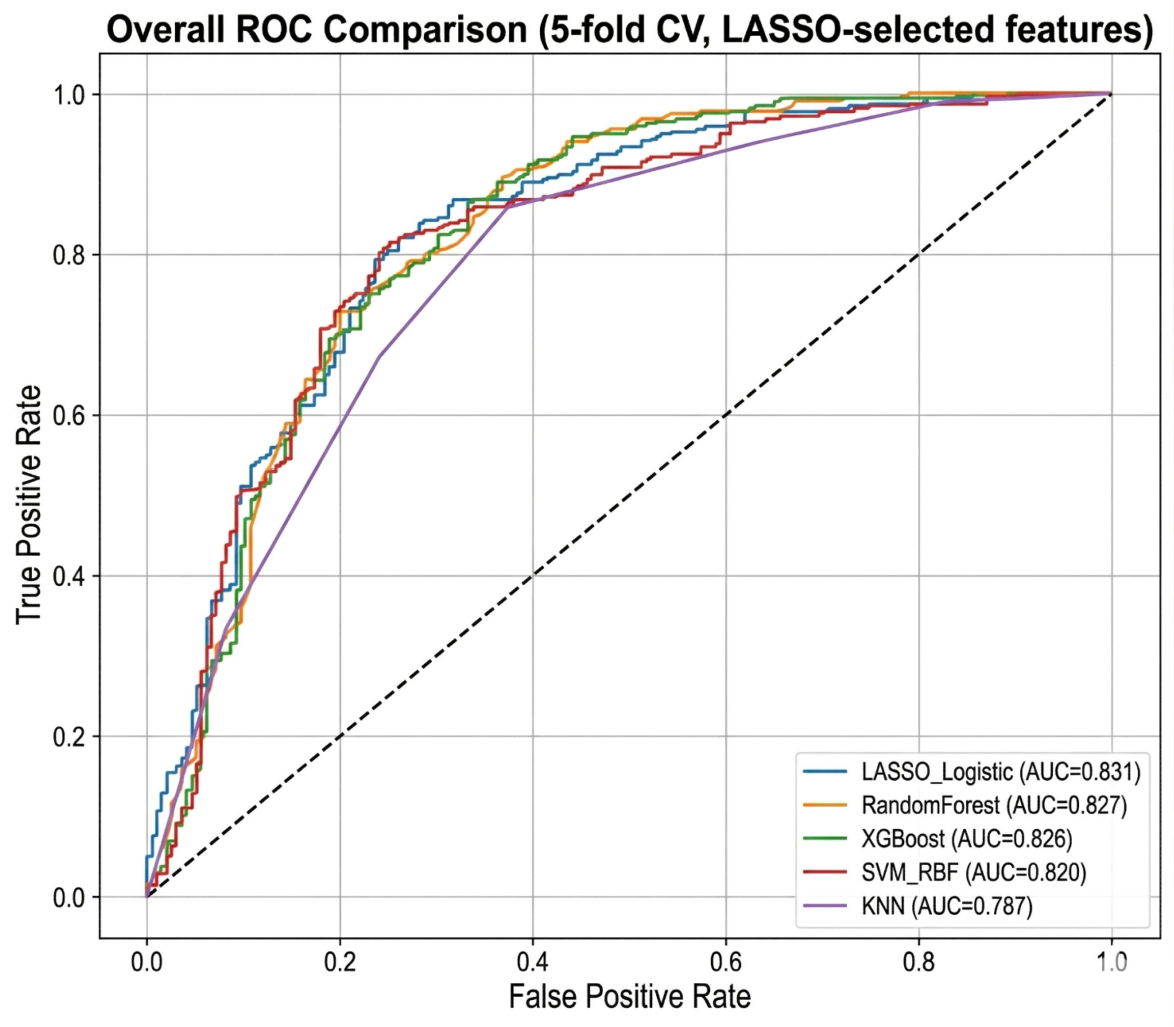
Overall ROC comparison of the five machine-learning classifiers evaluated using five-fold cross-validation with LASSO-selected features. The figure illustrates the trade-off between sensitivity and specificity for each model. The LASSO logistic regression model demonstrated the highest overall discrimination, while Random Forest and XGBoost achieved similar but slightly lower performance. The SVM-RBF and KNN classifiers exhibited the lowest discrimination among the models.

The Random Forest and XGBoost models demonstrated similar performance (AUCs = 0.827 and 0.826, respectively), whereas the SVM-RBF model showed slightly lower discrimination (AUC=0.820). The KNN classifier yielded the lowest performance (AUC=0.787). **Figure 2** displays the combined receiver-operating-characteristic (ROC) curves for all five classifiers, illustrating consistent behavior across folds and confirming that the LASSO model provided the highest overall discrimination. Random Forest and XGBoost followed closely, while SVM-RBF and KNN showed comparatively reduced performance. Precision-recall curves further demonstrated the high precision of the LASSO model in identifying hypertensive cases.

Class-wise performance metrics for the LASSO model are shown in **Table 2**. The model achieved an F1 score of 0.73 for the ≤120 mmHg group and 0.83 for the ≥140 mmHg group, with macro- and weighted-average F1 scores of 0.78–0.79. These findings indicate balanced precision and recall across SBP categories, confirming that the model not only identifies hypertensive participants effectively but also maintains reasonable sensitivity for normotensive individuals.

The table presents precision, recall, and F1-score for the two SBP categories (≤120 mmHg and ≥140 mmHg), illustrating balanced performance across groups. Macro- and weighted-average metrics are included to account for class imbalance.

Bootstrap resampling (n = 2,000) produced sTable 95% confidence intervals for AUC estimates across all classifiers, with no overlap suggesting instability. Overall, the LASSO model achieved the most favorable balance between performance and interpretability and was therefore selected as the primary model for downstream feature-importance analysis.

### 3.3 Feature importance and model interpretation

**Figure 3:**
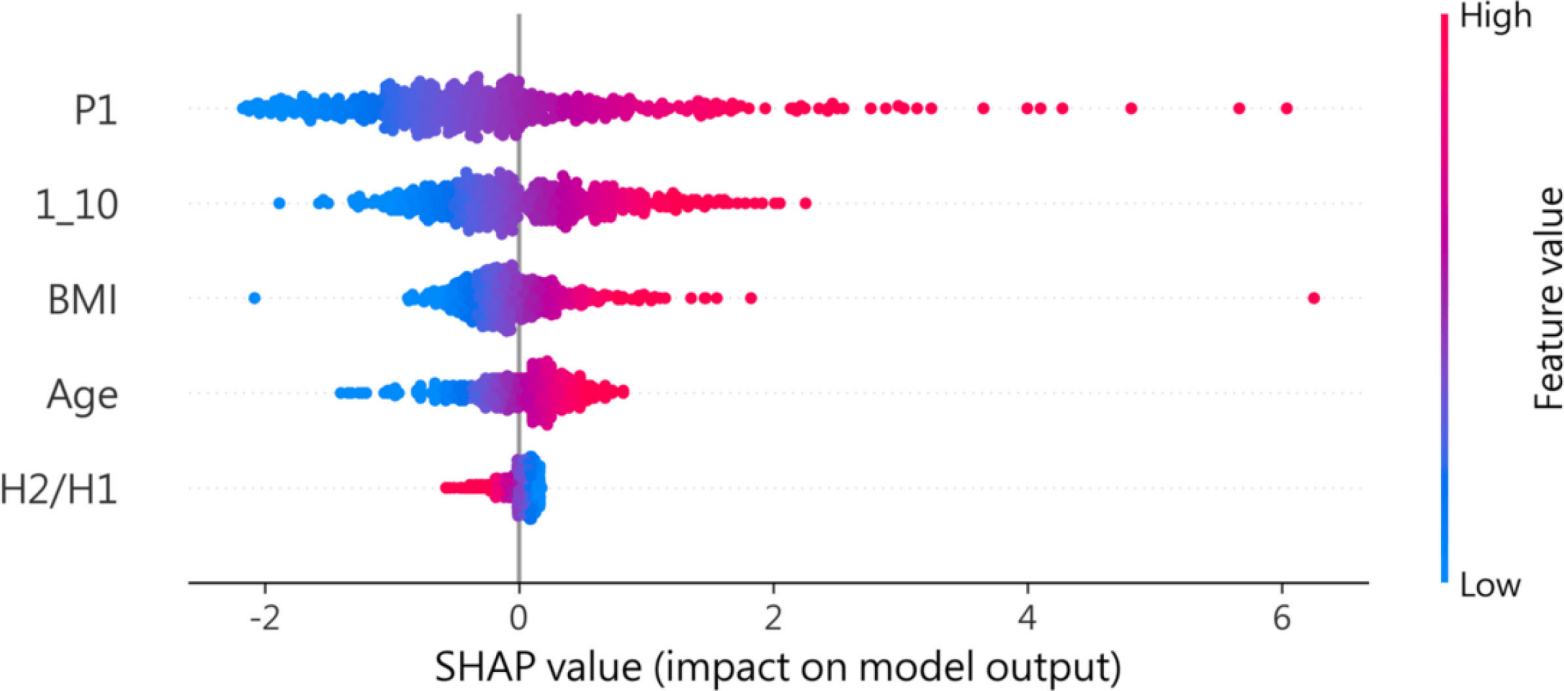
SHAP summary plot of the LASSO logistic regression model. Higher values of P1, 1_10, and BMI increased the predicted probability of hypertension, whereas lower values of H2/H1 were associated with elevated systolic pressure. Feature value is encoded by color from low (blue) to high (red).

The LASSO-logistic model identified five key predictive features, including age, BMI, waveform sharpness (1_10), systolic amplitude (P1), and harmonic ratio (H2/H1). Together, these predictors accounted for most of the model’s discriminative capacity. Among these variables, two PPG-derived features (P1 and 1_10) ranked among the most influential predictors alongside demographic variables such as age and BMI. **Figure 3** presents the SHAP summary plot of the LASSO model and illustrates the marginal contribution and directional influence of each selected feature on the classification of systolic blood-pressure readings.

Among these predictors, P1 and 1_10 produced the largest positive SHAP values, indicating that greater systolic amplitude and sharper waveform morphology were associated with an increased likelihood of elevated SBP. In contrast, H2/H1 showed a distinctly negative SHAP pattern, suggesting that reduced harmonic reflection corresponded to higher vascular load and diminished arterial compliance. Age and BMI also contributed positive effects, consistent with their well-established associations with arterial stiffness and BP. Taken together, these findings suggest that PPG waveform features provide complementary physiological information beyond basic anthropometric variables, reinforcing the interpretability of the LASSO-selected predictors and supporting their potential applicability as transparent, noninvasive digital biomarkers for assessing vascular load. While pulse rate variability (PRV) parameters derived from PPG share similarities with heart rate variability (HRV) metrics obtained from ECG, PPG provides additional information related to peripheral vascular dynamics. In particular, waveform-derived morphological features, such as systolic amplitude (P1), waveform sharpness (1_10), and harmonic ratios (H2/H1), reflect vascular compliance and pulse transmission characteristics that are not directly accessible from ECG signals. Therefore, the contribution of PPG extends beyond variability measures alone and includes physiologically meaningful vascular features relevant to BP regulation.

### 3.4 Overall model comparison

A comprehensive comparison of all classifiers is summarized in **Table 3**. Although ensemble-based methods (i.e., Random Forest and XGBoost) produced AUC values similar to the LASSO model, their interpretability was more limited. In contrast, the LASSO model offered a sparse and transparent representation of the predictive landscape, enabling direct physiological mapping of each selected feature. Consistent with the patterns shown in **Figure 2**, the overall ROC behavior across classifiers supported the robustness of the LASSO-based framework when applied to prescreened, high-quality PPG signals (SQI ≥0.2%). Taken together, these results demonstrate that a compact and physiologically interpretable set of PPG-derived features can achieve strong discriminatory performance, reinforcing the feasibility of PPG-based digital biomarkers for noninvasive blood-pressure assessment.

**Table 3:** Summary of overall model performance across the five classifiers.

Model	AUC	AUC_CI_95	AP	Best_Threshold	Accuracy	F1	Cohen_d	Cohen_d_CI_95
LASSO_Logistic	0.83085	[0.794, 0.868]	0.865375	0.426365	0.789063	0.828025	1.386227	[1.155, 1.640]
RandomForest	0.827372	[0.786, 0.867]	0.838316	0.49	0.794922	0.843982	1.441501	[1.217, 1.706]
XGBoost	0.825673	[0.786, 0.865]	0.838494	0.429048	0.789063	0.835366	1.394737	[1.153, 1.663]
SVM_RBF	0.820497	[0.781, 0.859]	0.840471	0.565175	0.789063	0.826923	1.382772	[1.147, 1.648]
KNN	0.787382	[0.746, 0.828]	0.810865	0.6	0.769531	0.821752	1.186054	[0.981, 1.421]

The table reports discrimination metrics (AUC and AP), optimal probability thresholds, classification accuracy, F1-scores, and Cohen’s d effect sizes with corresponding 95% confidence intervals. Although ensemble-based models achieved performance similar to the LASSO classifier, the LASSO model offered comparable accuracy alongside superior interpretability, supporting its use for downstream feature analysis.

### 3.5 Additional analysis of heart-rate influence on P1

To further investigate the physiological influence of heart rate on P1, an additional analysis was performed. Participants were stratified into low and high heart-rate groups based on quartiles. As shown in **Table 4**, P1 values were lower in the high heart-rate group compared with the low heart-rate group (mean: 508.6 vs 603.3; median: 490.1 vs 551.7). This difference was statistically significant (Mann–Whitney U=9619, p = 0.016), indicating that P1 is influenced by heart-rate-dependent physiological conditions.

**Table 4:** Comparison of P1 between low and high heart-rate groups.

Variable	Low HR (≤25th) (n=128)	High HR (≥75th) (n=128)	p-value	Effect size (r)
P1 (mean ± SD)	603.28 ± 318.22	508.59 ± 263.43	0.016	-0.17
P1 (median, IQR)	551.73 (408.01–729.97)	490.08 (344.23–646.69)		

**Figure 4:**
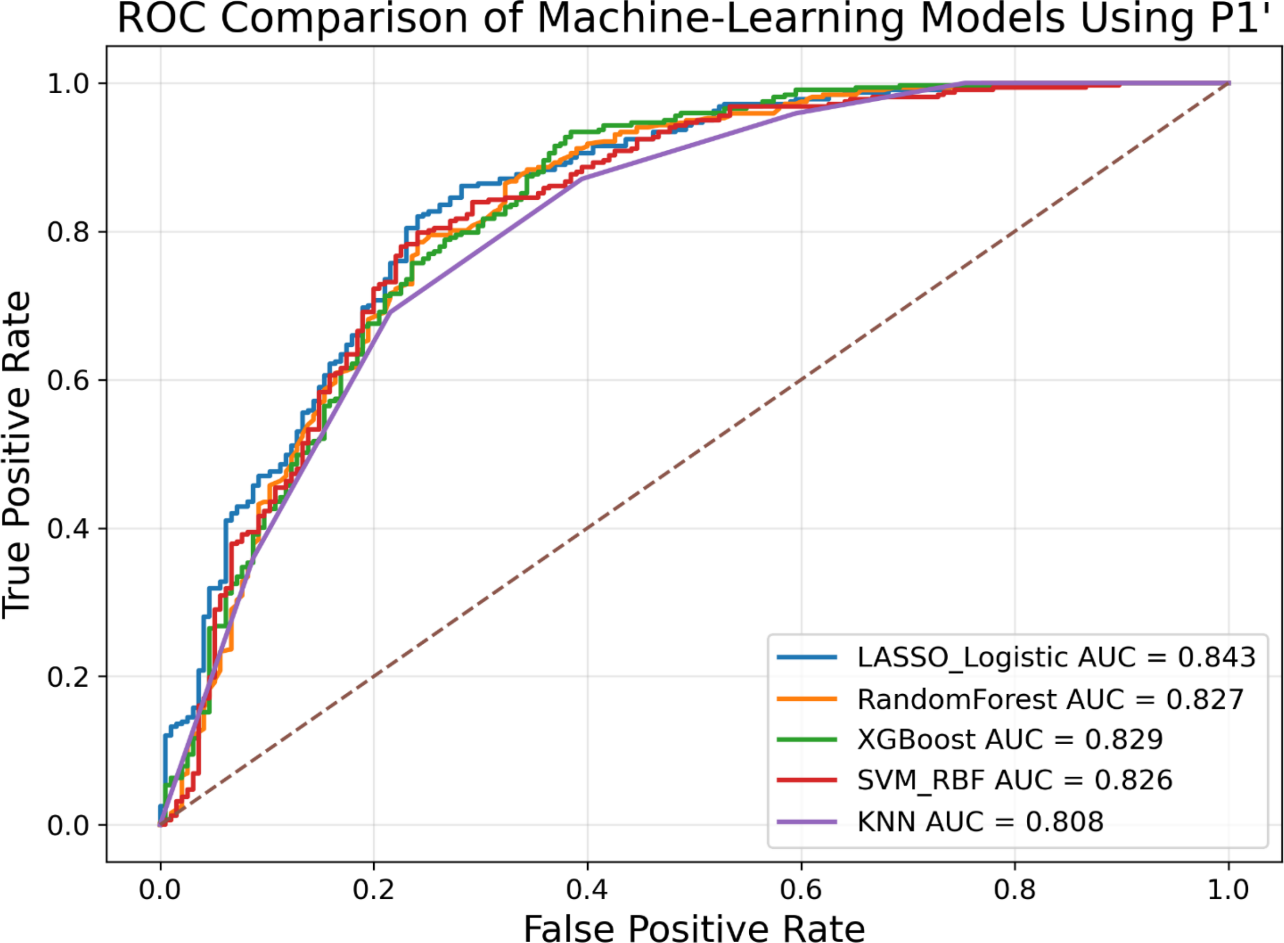
Overall ROC comparison of the five machine-learning classifiers evaluated using five-fold cross-validation with LASSO-selected features, using the heart-rate–adjusted parameter P1′. P1′ was defined as ln(P1/pulse rate) to account for the nonlinear physiological influence of heart rate on pulse waveform characteristics. The figure illustrates the trade-off between sensitivity and specificity for each model. The LASSO logistic regression model demonstrated the highest overall discrimination, while Random Forest and XGBoost achieved comparable but slightly lower performance. The SVM-RBF and KNN classifiers exhibited the lowest discrimination among the models.

To explore this effect within the modeling framework, we introduced a heart-rate-adjusted parameter defined as P1′ = ln(P1/heart rate). When substituting P1 with P1′, model performance remained comparable, with slight numerical improvement (e.g., AUC 0.831 vs 0.843 in the LASSO model). The corresponding ROC curves using P1′ are shown in **Figure 4**.

## 4 Discussion

### 4.1 Principal findings

In this study, we developed a compact and physiologically interpretable machine-learning framework for rapid hypertension screening using PPG signals. By combining signal-quality–filtered PPG waveforms with LASSO-based feature selection, the resulting logistic regression model achieved strong discriminatory performance (AUC=0.83, 95% CI 0.79–0.87) while maintaining clinical transparency and computational simplicity. Importantly, statistically significant differences in individual variables (e.g., age and BMI) do not necessarily guarantee optimal classification performance, highlighting the need for a multivariable modeling approach.

Among the evaluated classifiers, the LASSO-logistic model provided the most favorable balance between predictive performance and interpretability. The model selected five key predictors, namely age, BMI, P1, 1_10, and H2/H1, from an initial set of thirty candidate features. These predictors collectively capture vascular stiffness, waveform morphology, and systemic determinants of hemodynamic load, thereby providing complementary physiological insights into systolic blood-pressure regulation.

Previous PPG-based studies have similarly explored waveform morphology and pulse-wave characteristics as indicators of BP and vascular stiffness (Karimpour et al., 2023). However, many earlier investigations primarily focused on correlation analyses between individual waveform features and BP rather than implementing predictive modeling frameworks (Gholamhosseini et al., 2018). In addition, signal-quality control has not always been explicitly incorporated into feature-selection pipelines in earlier studies. By integrating SQI waveform selection with interpretable feature reduction, the present framework extends previous physiological investigations toward a practical predictive model for hypertension screening.

The combined use of SQI-based signal screening and LASSO-based dimensionality reduction establishes a scalable structure for clinical or community-level hypertension triage. These findings suggest that high-quality single-sensor PPG signals, even without ECG or pulse-transit-time calibration, may provide a practical and resource-efficient approach for preliminary hypertension screening.

The observed model performance should be interpreted in the context of current hypertension screening practices. Conventional cuff-based BP measurement remains the clinical reference standard for diagnosing hypertension; however, its effectiveness in opportunistic outpatient screening can be influenced by measurement variability, white-coat effects, and operational constraints. Previous literature has reported that the sensitivity of hypertension screening strategies varies widely, ranging from approximately 51% to 91% (Pavlik et al., 2000; Ostchega et al., 2010; Lim et al., 2014), depending on the measurement protocol and reference standard used. In this context, the discriminative performance observed in the present study (AUC = 0.83) suggests that PPG-derived waveform features may provide a practical adjunct for scalable hypertension risk screening, particularly in settings where continuous or opportunistic monitoring is desirable. Because the proposed model is intended as a screening tool rather than a diagnostic substitute, individuals classified as hypertensive should undergo confirmatory BP assessment, whereas repeated screening may help detect progression in borderline cases.

### 4.2 Physiological and clinical interpretation of key features

The five predictors selected by the LASSO-logistic model—P1, 1_10, H2/H1, age, and BMI—collectively capture vascular and systemic determinants of blood-pressure regulation. Each feature reflects a distinct physiological mechanism related to arterial compliance, wave reflection, or overall hemodynamic load.

#### 4.2.1 P1 (systolic amplitude) is an indicator of stroke volume and peripheral resistance

P1, the apex of the percussion wave, represents the forward-traveling pressure wave generated by left ventricular ejection and reflects the propagation of the ventricular pressure wave through the arterial system. Its magnitude and timing are influenced by the interaction between left ventricular contractile function, vascular tone, systemic vascular resistance, and heart rate. In the present study, the systolic peak amplitude (P1), measured relative to baseline, was higher in participants with elevated resting SBP.

The observed increase in P1 amplitude among hypertensive individuals can be physiologically attributed to the ejection of stroke volume into a less compliant arterial system together with reduced Windkessel buffering capacity. Increased arterial stiffness augments left ventricular afterload and results in a greater instantaneous rise in arterial pressure during systole, thereby elevating the systolic peak of the PPG waveform (Hersant et al., 2025). Similar relationships between peripheral pulse amplitude and elevated arterial pressure have also been reported in previous pulse-wave analyses (Elgendi et al., 2024).

Importantly, the amplitude of P1 is not solely determined by vascular stiffness or ventricular ejection, but is also modulated by heart rate–dependent physiological conditions (Wilkinson et al., 2000). At higher heart rates, shortened diastolic filling time reduces ventricular preload and stroke volume, whereas lower heart rates allow greater diastolic filling and increased preload (Schaefer et al., 1988; Cheng et al., 1990). These nonlinear changes in loading conditions influence the magnitude of the forward-traveling pressure wave and may confound the interpretation of P1 when used as a standalone parameter (Merillon et al., 1983).

In response to this physiological consideration, we introduced a heart-rate–adjusted parameter, P1′, defined as ln(P1/pulse rate). This transformation was designed to account for the nonlinear relationship between pulse waveform amplitude and heart rate, rather than assuming a linear dependency. The logarithmic scaling reflects the concept that relative changes in physiological loading conditions, rather than absolute differences, are more relevant to waveform morphology. Incorporation of P1′ into the modeling framework preserved or slightly improved discriminative performance, suggesting that adjusting for heart rate–dependent effects enhances the physiological interpretability and robustness of P1-related features.

#### 4.2.2 1_10 (waveform sharpness) is a surrogate of arterial stiffness and reflection timing

Peripheral PPG waveforms are shaped by fundamental hemodynamic processes linking arterial structure to BP (Matti Huotari et al., 2011). When the left ventricle ejects blood, a forward-traveling pressure wave propagates through the arterial tree. As the wave encounters impedance sites such as arteriolar branching points or microvascular beds, part of the energy is reflected. The superposition of forward and reflected waves produces the composite peripheral pulse waveform observed in arteries including the radial or femoral artery (Hashimoto and Ito, 2010). The timing and amplitude of these waveform components are governed by arterial stiffness and compliance, as described by the Moens–Korteweg equation, which relates pulse-wave velocity to vessel elasticity and structural properties (von Wowern et al., 2017). In hypertension, elevated arterial stiffness accelerates PWV, causing the reflected wave to return earlier, often during systole rather than diastole (Chen et al., 2005).

In this context, the waveform sharpness parameter (1_10) quantifies the relative width of the systolic waveform at a predefined fractional amplitude level (i.e., 1/10 of the peak height). A narrower width at this level reflects a steeper systolic upstroke and a more rapid rise in arterial pressure. Importantly, earlier return of reflected waves in stiffened arteries leads to increased systolic augmentation and compression of the systolic waveform, thereby reducing its temporal width. This morphological change is consistent with increased arterial stiffness and altered wave reflection timing. Thus, 1_10 captures the combined effects of vascular compliance and reflection timing on waveform geometry, as described in our previous work (Lin et al., 2025).

Consequently, morphological features of the PPG waveform provide physiologically meaningful indicators of vascular load and arterial stiffness. Similar waveform-geometry parameters have been reported as markers of vascular compliance and pulse-wave reflection timing in previous PPG waveform studies (Szoltysek-Boldys et al., 2024; Song et al., 2025).

#### 4.2.3 H2/H1 (harmonic ratio) acts as a marker of wave reflection and compliance loss

Elevated BP accelerates pulse-wave velocity and leads to the earlier return of reflected waves from peripheral arteries. This premature overlap between forward and reflected waves modifies the contour of the peripheral pulse waveform and alters its frequency-domain characteristics. In particular, the attenuation of higher harmonic components reflects diminished arterial compliance and altered vascular impedance (Boonya-Ananta et al., 2021).

Harmonic-analysis theory proposes that vascular beds behave as frequency-selective resonators, in which each harmonic component of the PPG signal reflects elastic and reflective properties of specific vascular segments. Suppression of the second and higher harmonics has been observed in older and hypertensive populations, reflecting impaired arterial compliance and altered wave propagation (Rensma et al., 2020).

Comparable reductions in higher-order harmonic content have also been described in studies applying harmonic analysis to arterial pulse waveforms in aging and hypertensive populations (Rensma et al., 2020; Song et al., 2025).

In this context, the H2/H1 ratio reflects the relative contribution of reflective waveform components to the fundamental cardiac-driven pulse. The first harmonic (H1) corresponds to the fundamental frequency of the PPG signal and primarily represents the cardiac cycle, whereas the second harmonic (H2) is associated with reflective components of the waveform, including the dicrotic notch and wave reflection phenomena. A reduction in H2/H1 indicates attenuation of reflective and higher-frequency components, which is consistent with increased arterial stiffness, altered vascular impedance, and diminished arterial compliance, as supported by prior harmonic analysis studies of PPG signals (Park et al., 2023).

#### 4.2.4 Age and BMI are systemic determinants of vascular load

BP is a highly heritable trait (Levy et al., 2000), and hundreds of independent genetic loci capable of influencing BP have been identified to date (International Consortium for Blood Pressure Genome-Wide Association, S, 2011; Keaton et al., 2024). Nonetheless, all reported loci together explain<10% of BP variance, underscoring the importance of additional factors and gene-environment interactions.

BP generally increases with age beginning in young adulthood, and lifetime risks for incident hypertension exceed 80% in US populations (Chen et al., 2019). Although this trend is often described as a normative aging pattern, it may not be inevitable. Recent data indicate that age-related increases in SBP and DBP may be avoided in young adults who maintain stable BMI during long-term follow-up into middle age (Lloyd-Jones et al., 2007).

The mechanisms underlying obesity and obesity-related hypertension are complex and often interdependent. Beyond genetic and environmental contributions, key roles are played by the sympathetic nervous system, renal and adrenal function, endothelial responses, adipokine secretion, and insulin resistance.

Human obesity and hypertension are associated with marked alterations in large-artery structure and function, including increased arterial stiffness and reduced arterial compliance and distensibility (Levy et al., 2008). Even more importantly, changes in small resistance arteries exert a critical influence, as these vessels contribute directly to the pathogenesis of ischemic cerebral events, coronary artery disease, and renal insufficiency (Levy et al., 2008), and carry strong prognostic significance in obesity-related conditions such as hypertension (Rizzoni et al., 2003).

Obesity and hypertension together are associated with increased morbidity and escalating medical costs, representing a major public health challenge. Effective control of both conditions within at-risk populations remains an urgent priority.

### 4.3 Comparison with previous studies

While prior PPG-based investigations have consistently reported correlations between waveform morphology and BP, relatively few have progressed toward predictive modeling that incorporates explicit signal-quality control. Classical works have focused on defining fiducial points of the pulse waveform, including the systolic peak, waveform foot, and dicrotic notch, in order to relate geometric parameters to vascular compliance and reflection timing (Takazawa et al., 1998; Elgendi, 2012). These studies established the physiological basis of pulse-wave analysis but were largely limited to descriptive correlations between waveform features and cardiovascular function. Subsequent research has explored a broader range of PPG-derived indices for blood-pressure estimation, including parameters such as the stiffness index (SI), reflection index (RI), augmentation-related measures, and harmonic waveform components, which have been proposed as indicators of vascular stiffness and hemodynamic load. More recently, machine-learning approaches have been applied to estimate or classify BP from PPG signals. For example, several studies have used neural-network–based frameworks to infer BP from raw or minimally processed PPG signals. However, many of these approaches rely on complex black-box models that offer limited physiological interpretability. In addition, methodological variability in PRV analysis and challenges in establishing consistent fiducial references across datasets have also been reported (Park et al., 2021).

Beyond academic studies, wearable devices have also begun to explore optical cardiovascular monitoring. Smartwatch platforms such as the Apple Watch have incorporated PPG sensing for cardiovascular health monitoring and may provide notifications related to potential cardiovascular abnormalities. Nevertheless, current consumer-grade implementations are primarily designed for risk screening or health alerts rather than precise blood-pressure quantification, reflecting the technical challenges associated with translating peripheral waveform morphology into accurate cuffless BP measurements.

Building on our previous work (Lin et al., 2025), which examined associations between comprehensive time- and frequency-domain PPG features and SBP, the present study shifts from physiological correlation to predictive modeling. We therefore implemented a SQI selection pipeline to ensure that only high-fidelity, artifact-free PPG segments contributed to model training and evaluation. Through LASSO-based feature reduction, the model distilled an initial collection of 30 variables into five clinically interpretable predictors, including age, BMI, P1, 1_10, and H2/H1, while maintaining strong discriminative performance (AUC=0.83).

By integrating signal-quality–controlled data selection with interpretable machine-learning modeling, the present framework differs from earlier PPG–BP studies that either relied on descriptive correlation analyses or adopted opaque black-box prediction models. This approach bridges classical pulse-wave physiology with modern digital cardiovascular screening and may contribute to the development of transparent and clinically interpretable cuffless blood-pressure monitoring strategies.

### 4.4 Methodological strengths

The primary methodological strength of this study lies in its simplicity and clinical applicability. Rather than positioning the absence of ECG or PTT measurements as the primary innovation, the strength of the present framework lies in combining single-channel PPG acquisition with interpretable feature selection and transparent machine-learning classification. This design markedly reduces the complexity, cost, and setup time associated with traditional multi-sensor systems, making it well suited for primary care and community-based health screening.

Furthermore, the integration of LASSO feature selection with explainable machine-learning methods ensures both computational efficiency and interpretability. By reducing 30 candidate variables to five physiologically meaningful predictors—age, BMI, P1, 1_10, and H2/H1—the model maintains strong performance (AUC=0.83) while preserving transparency in how each feature contributes to blood-pressure discrimination. This compact and interpretable structure supports real-world clinical translation and aligns with regulatory expectations for trustworthy AI in digital-health applications.

In summary, the proposed framework represents a rapid, noninvasive, and interpretable PPG-based tool capable of identifying individuals at risk of hypertension without requiring concurrent ECG or arterial pressure reference signals. This methodological minimalism enhances its suitability for large-scale deployment in outpatient and telehealth environments.

### 4.5 Limitations

Several limitations should be acknowledged. First, this study was conducted in a single-center, cross-sectional cohort, and all measurements were obtained under resting outpatient conditions. As a result, the model’s generalizability to ambulatory or home-monitoring environments remains uncertain. Future studies should incorporate more diverse populations and multi-site datasets to ensure robustness across different demographic and clinical contexts.

Second, the classification framework dichotomized SBP into two categories (≤120 mmHg vs ≥140 mmHg), a choice that aids clinical interpretability but may obscure transitional patterns in prehypertensive individuals. A continuous prediction strategy or regression-based modeling may further improve the precision of BP estimation.

Third, despite its strong predictive performance, PPG is inherently sensitive to peripheral vascular tone, skin temperature, and motion artifacts (Allen, 2007). Although data acquisition was performed under controlled clinical conditions, future deployment in ambulatory settings will require adaptive signal-stabilization or quality-monitoring algorithms to maintain reliability.

Finally, the present study focused exclusively on SBP classification and did not incorporate diastolic BP measurements. Although systolic pressure is closely linked to arterial stiffness and waveform morphology captured by PPG signals, the absence of DBP assessment may limit the detection of isolated diastolic hypertension. Future studies incorporating both systolic and diastolic parameters may further improve the comprehensiveness of PPG-based hypertension screening.

### 4.6 Future directions

Building on the present findings, future work should examine continuous, real-time hypertension screening using wearable or mobile-integrated PPG devices. The simplicity of the single-sensor design allows seamless incorporation into smartwatches, fingertip probes, or smartphone cameras, potentially extending hypertension surveillance beyond traditional clinical settings.

In addition, integrating PPG-derived indices with clinical risk factors and longitudinal data could enable more individualized risk stratification and treatment monitoring. Combining this interpretable framework with adaptive or deep learning models may further enhance predictive accuracy while maintaining transparency.

Finally, prospective validation in larger, multi-ethnic cohorts, together with correlation against gold-standard oscillometric or intra-arterial measurements, will be essential for establishing this approach as a scalable, low cost, front-line digital biomarker for population-level hypertension screening.

In addition, while ECG acquisition is increasingly feasible in modern wearable systems, the present study intentionally focused on a single-sensor PPG approach to reduce system complexity and enhance scalability for community-based screening. Future work may explore hybrid PPG–ECG models to evaluate potential performance improvements. While PTT may serve as a physiological surrogate of vascular aging, the present study intentionally focuses on a single-sensor PPG framework to maintain simplicity and clinical feasibility in routine outpatient settings. Future studies may explore whether PTT-based approaches provide additional value beyond single-sensor PPG models.

Furthermore, extending the current binary classification framework to multiclass or continuous prediction models may improve characterization of intermediate blood-pressure states. Future studies may also evaluate whether frequency-domain features, such as H1 or normalized harmonic sums, can replace time-domain amplitude or sharpness metrics while improving robustness to noise.

Importantly, nonlinear relationships between pulse waveform features and physiological variables should be considered. In the present study, we observed that P1 is influenced by heart-rate-dependent physiological conditions, likely reflecting changes in diastolic filling and preload. A simple logarithmic transformation (P1′ = ln(P1/heart rate)) was applied to partially account for these nonlinear effects, and yielded comparable or slightly improved model performance. These findings suggest that incorporating physiologically motivated transformations may enhance feature interpretability while maintaining predictive performance.

Validation against intra-arterial blood pressure measurements in selected clinical settings may further strengthen the translational potential of this approach, although such validation remains constrained by its invasive nature.

## 5 Conclusion

This study demonstrates that a compact and interpretable model using only five predictors, including age, BMI, P1, 1_10, and H2/H1, can effectively distinguish hypertensive from normotensive individuals without the need for auxiliary sensors such as ECG or arterial pressure transducers. By relying solely on a single-channel PPG signal together with basic demographic inputs, the framework enables rapid, noninvasive, and low-cost preliminary hypertension screening suitable for outpatient and broader community settings. From a clinical standpoint, the proposed model can function as an early-warning digital biomarker, helping identify individuals who require confirmatory blood-pressure measurement or further cardiovascular evaluation. Its simplicity supports seamless integration into portable or wearable devices, allowing opportunistic screening in resource-limited or telemedicine environments.

Moreover, the model’s explainable structure, built on physiologically interpretable features rather than opaque latent representations, enhances clinical trust and facilitates regulatory alignment. The convergence of physiological insight, algorithmic transparency, and practical deployability positions this framework as a scalable bridge between signal-level PPG analytics and real-world hypertension prevention programs.

## Data Availability

The raw data supporting the conclusions of this article will be made available by the authors, without undue reservation.
